# County-Level Association Between Social Vulnerability and Rheumatoid Arthritis-Related Mortality in the United States

**DOI:** 10.3390/medsci14020314

**Published:** 2026-06-12

**Authors:** Wan-Ying Lin, Yu-Che Lee, Abira A. Chowdhury, Linda M. Burns, Hsin-Yao Wang

**Affiliations:** 1 Department of Medicine, Jacobs School of Medicine, University at Buffalo, Buffalo, NY 14260, USA; wlin225@buffalo.edu (W.-Y.L.);; 2 Department of Internal Medicine, Catholic Health System, Buffalo, NY 14214, USA; 3 Department of Pulmonary and Critical Care Medicine, University at Buffalo, Buffalo, NY 14260, USA; yuchelee@buffalo.edu; 4 Buffalo Rheumatology and Medicine, Buffalo, NY 14226, USA; 5 School of Medicine, National Tsing Hua University, Hsinchu 300044, Taiwan; 6 20/20 Biolabs, Gaithersburg, MD 20877, USA

**Keywords:** rheumatoid arthritis, social vulnerability index, mortality, county-level analysis

## Abstract

**Objectives:** To evaluate associations between social vulnerability and rheumatoid arthritis (RA)-related mortality in the United States, with emphasis on domain-specific effects of the Social Vulnerability Index (SVI). **Methods:** We conducted a county-level ecological study of RA-related mortality from 2010 to 2019 using age-adjusted mortality rates and the Centers for Disease Control and Prevention SVI. Gamma regression models examined associations between RA mortality and overall SVI and four thematic domains, including socioeconomic status, household composition and disability, minority status and language, housing type and transportation by using both continuous and quartile-based measures. **Results:** Between 2010 and 2019, 354,280 deaths occurred among individuals with RA, corresponding to a mean age-adjusted mortality rate of 9.7 per 100,000 population. In multivariable analyses adjusting for all SVI domains, household composition and disability vulnerability demonstrated the strongest and most consistent positive association with mortality, with a dose–response relationship across quartiles. Housing type and transportation vulnerability showed a modest positive association. Minority status and language vulnerability was inversely associated with mortality, whereas socioeconomic vulnerability was not significant in continuous models but demonstrated an inverse association with mortality in quartile-based analyses. **Conclusions:** RA mortality is differentially associated with specific domains of social vulnerability rather than overall vulnerability burden. Household composition and disability represent clinically salient risk factors, demonstrating the relevance of functional status and caregiving context in RA outcomes. Domain-specific assessment of social vulnerability may enhance clinical risk stratification and inform more targeted, patient-centered RA management.

## 1. Introduction

Rheumatoid arthritis (RA) is a chronic systemic autoimmune disease associated with increased morbidity and mortality compared with the general population. Excess mortality in RA is driven by disease-related complications, including interstitial lung disease and cardiovascular disease, particularly coronary artery disease [1,2,3,4]. Recent U.S. analyses using CDC WONDER data indicate that RA-related mortality has declined over the past two decades but remains clinically meaningful. One study reported that the age-adjusted RA mortality rate decreased from 5.65 per 100,000 population in 1999 to 3.33 per 100,000 in 2019, followed by an increase to 4.07 per 100,000 in 2020 [5]. Established biological risk factors for poor outcome include older age at diagnosis, male sex, seropositivity, and persistently high disease activity [6,7,8]. However, these factors do not fully explain the persistent mortality gap observed in RA, suggesting that non-biological contributors may also play an important role. Non-biological determinants, including social determinants of health, have been increasingly recognized as important drivers of RA outcomes. Socioeconomic position, employment status, and race/ethnicity are associated with functional outcomes, disease progression, and mortality in RA [9,10,11]. For example, Canadian registry data indicate that full-time employment, higher income, and White race are associated with greater improvements in physical function over 12 months, whereas smoking predicts smaller gains [10]. Similarly, national U.S. data demonstrate progressively worse functional outcomes and a higher likelihood of functional decline among individuals in lower socioeconomic groups [12]. International evidence supports these findings: a population-based study in Taiwan found that individual socioeconomic status was strongly associated with functional outcomes and mortality [13].

Social vulnerability may influence RA mortality through multiple, interrelated pathways. These include delayed diagnosis, reduced access to rheumatology specialty care, differential use and persistence of disease-modifying antirheumatic drugs (DMARDs), and higher burdens of comorbid conditions. In addition, limited social support, caregiver availability, and functional assistance may impair effective management of chronic disability, particularly among patients with advanced disease or coexisting physical limitations [14]. Area-level social factors may therefore shape disease outcomes not only through healthcare access, but also through broader influences on treatment continuity, self-management capacity, and resilience to acute illness [15].

Despite increasing recognition of the importance of social determinants in RA, nationwide U.S. studies examining the association between area-level social vulnerability and RA-specific mortality remain limited. The Centers for Disease Control and Prevention Social Vulnerability Index (SVI), which aggregates 15 social variables across four domains—socioeconomic status; household composition and disability; minority status and language; and housing type and transportation—provides a comprehensive framework for capturing multidimensional social risk at the community level. While higher overall social vulnerability is generally associated with adverse health outcomes, the relationship between individual SVI domains and disease-specific mortality may vary.

Importantly, the study period coincides with major changes in healthcare access in the United States, including expansions in insurance coverage beginning in 2010. Prior studies have shown that these changes were associated with improved access to preventive and specialty care and reductions in all-cause mortality, particularly among low-income populations [16,17,18]. Such shifts in healthcare access may differentially influence observed mortality patterns across socioeconomic strata and provide important context for interpreting heterogeneous associations between social vulnerability domains and RA mortality. 

In this study, we used national U.S. mortality data from 2010 to 2019 to examine the association between the Centers for Disease Control and Prevention Social Vulnerability Index and RA–related mortality at the county level, with particular emphasis on domain-specific effects. By evaluating heterogeneity across multiple dimensions of social vulnerability, this analysis seeks to better characterize the role of social context in shaping population-level RA mortality and to inform future efforts aimed at reducing persistent disparities in RA outcomes.

## 2. Materials and Methods

### 2.1. Data Sources and Study Design

We conducted an ecological study to evaluate county-level associations between social vulnerability and RA-related mortality in the United States from 2010 through 2019. County-level crude and age-adjusted RA mortality rates were obtained from the Centers for Disease Control and Prevention Wide-ranging Online Data for Epidemiologic Research (CDC WONDER) Multiple Cause of Death database. Deaths were identified using International Classification of Diseases, Tenth Revision (ICD-10) codes M05.0–M06.9, in which RA was recorded as a cause of death. Mortality records flagged as unreliable, suppressed, missing, or non-numeric were excluded prior to analysis.

County-level social vulnerability was assessed using the Centers for Disease Control and Prevention Social Vulnerability Index (SVI), which provides percentile rankings for an overall composite index (RPL_THEMES) and four thematic domains: socioeconomic status (RPL_THEME1), household composition and disability (RPL_THEME2), minority status and language (RPL_THEME3), and housing type and transportation (RPL_THEME4). Higher percentile values indicate greater social vulnerability. Mortality and SVI data were linked deterministically using county Federal Information Processing Standards (FIPS) codes.

The primary outcome was the county-level age-adjusted RA-related mortality rate. The primary exposures were the overall SVI percentile ranking and the percentile rankings of the four SVI thematic domains. SVI measures were analyzed both as continuous percentile rankings and as quartile-based categories, with the lowest quartile representing counties with the least social vulnerability.

### 2.2. Ethics Statement

The study was conducted using publicly available, de-identified county-level data from the U.S. Centers for Disease Control and Prevention. As no identifiable human subjects were involved, the study was exempt from institutional review board review in accordance with U.S. federal regulations governing human subjects research.

### 2.3. Statistical Analysis

All statistical analyses were conducted using R statistical software (version 4.3.1; R Foundation for Statistical Computing, Vienna, Austria). Given the continuous, positive, and right-skewed distribution of mortality rates, generalized linear models with a Gamma distribution and log link function were used throughout. Model coefficients were exponentiated and interpreted as rate ratios with corresponding 95% confidence intervals. Statistical significance was evaluated using two-sided tests with an alpha level of 0.05. To assess potential multicollinearity among the four SVI thematic domains included in the multivariable model, variance inflation factors (VIFs) were calculated. Pairwise Pearson correlations among the four domains were also examined to characterize the degree of overlap among SVI domains.

### 2.4. Univariable Analysis

In the first analytic stage, univariable Gamma regression models were fitted to evaluate the independent association between each social vulnerability measure and RA mortality. Separate models were constructed for the overall SVI and for each of the four SVI thematic domains. In these models, SVI measures were treated as continuous percentile rankings to estimate the relative change in mortality associated with increasing social vulnerability.

### 2.5. Multivariable Analysis

In the second analytic stage, multivariable Gamma regression models were used to assess the joint associations between social vulnerability domains and mortality. A primary multivariable model included all four SVI thematic domains simultaneously, allowing estimation of their mutually adjusted associations with RA mortality. Exponentiated coefficients from this model represent the mutually adjusted association of each domain with mortality after accounting for the remaining domains. A forest plot was generated to visually summarize effect estimates from multivariable models.

### 2.6. Univariable and Multivariable Quartile-Based Analysis

In the third analytic stage, social vulnerability measures were categorized into quartiles based on their county-level distributions to evaluate potential non-linear and threshold effects. Quartile-based Gamma regression models were constructed using the lowest quartile (the least vulnerable group) as the reference category. Both univariable and multivariable quartile-based models were fitted, including a model examining the overall SVI quartiles and a model incorporating quartiles of the four SVI thematic domains simultaneously.

## 3. Results

### 3.1. Mortality Burden and Study Population

Between 2010 and 2019, a total of 354,280 deaths were recorded among individuals with RA in the United States. Across the study period, the mean age-adjusted RA–related mortality rate was 9.7 per 100,000 population. After exclusion of counties with unreliable, suppressed, or missing mortality estimates, the final analytic dataset included counties with complete mortality and social vulnerability data for subsequent regression analyses.

### 3.2. Univariable Associations of Continuous SVI Measures with RA Mortality

In univariable Gamma regression analyses, associations between social vulnerability and RA mortality varied across SVI domains (Table 1). Household composition and disability vulnerability (RPL_THEME2) was positively associated with mortality, with higher vulnerability corresponding to increased mortality rates (exp(coef) 1.14, 95% CI 1.09–1.19, *p* < 0.001). In contrast, greater minority status and language vulnerability (RPL_THEME3) was associated with lower mortality (exp(coef) 0.90, 95% CI 0.86–0.94, *p* < 0.001).

The overall SVI was not significantly associated with mortality in univariable analysis (exp(coef) 1.00, 95% CI 1.00–1.00, *p* = 0.205). Similarly, socioeconomic vulnerability showed no significant association with mortality (exp(coef) 1.00, 95% CI 1.00–1.00, *p* = 0.208). Housing type and transportation vulnerability were also not significantly associated with mortality in univariable models (exp(coef) 1.02, 95% CI 0.98–1.07, *p* = 0.367).

### 3.3. Multivariable Associations of Continuous SVI Measures with RA Mortality

**Figure 1:**
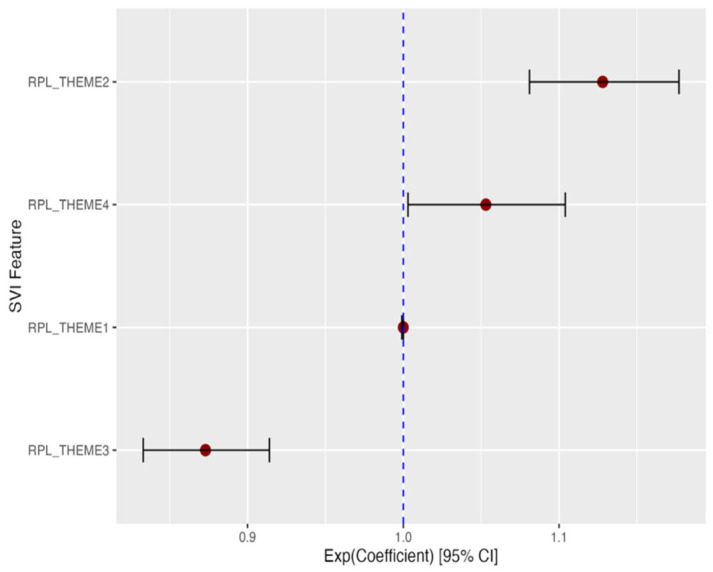
The forest plot shows the exponentiated coefficients with 95% confidence intervals for each variable in the multivariable analysis. Values to the right of 1.0 indicate increased odds, while values to the left indicate decreased odds. The CDC SVI includes four domains: socioeconomic status (RPL_THEME1), household composition & disability (RPL_THEME2), minority status & language (RPL_THEME3), and housing type & transportation (RPL_THEME4).

In multivariable Gamma regression models simultaneously accounting for all four SVI thematic domains, distinct and divergent patterns of association were observed (Figure 1). Household composition and disability vulnerability (RPL_THEME2) remained a robust associated factor of county-level RA mortality, with each unit increase associated with a 13% higher mortality rate (exp(coef) 1.13, 95% CI 1.08–1.18, *p* < 0.001). Minority status and language vulnerability (RPL_THEME3) was inversely associated with mortality, with higher vulnerability in this category corresponding to lower mortality (exp(coef) 0.87, 95% CI 0.83–0.91, *p* < 0.001). Housing type and transportation vulnerability (RPL_THEME4) showed a modest but statistically significant positive association with mortality (exp(coef) 1.05, 95% CI 1.00–1.10, *p* = 0.037). By contrast, socioeconomic vulnerability was not significantly associated with mortality in the multivariable model (exp(coef) 1.00, 95% CI 1.00–1.00, *p* = 0.174).

Multicollinearity diagnostics supported the stability of the multivariable model. VIF values were low for all four SVI domains: RPL_THEME1 = 1.002, RPL_THEME2 = 1.052, RPL_THEME3 = 1.203, and RPL_THEME4 = 1.253. Pairwise correlations among domains were generally weak, with the highest correlation observed between RPL_THEME3 and RPL_THEME4 (r = 0.400), followed by RPL_THEME2 and RPL_THEME4 (r = 0.201) (Supplementary Table S1). These findings suggest that multicollinearity among the SVI domains was limited and unlikely to materially affect the mutually adjusted estimates.

When evaluated jointly, the results showed that household composition and disability and minority/language vulnerabilities exert the strongest and most consistent effects on RA mortality, while the other domains demonstrate weaker or non-significant associations.

### 3.4. Univariable Associations of SVI Quartiles with RA Mortality

Univariable analyses using quartile-based categorizations of the SVI domains revealed heterogeneous associations with RA mortality (Table 2). Household composition and disability vulnerability (RPL_THEME2) showed a clear positive relationship with mortality. Counties in the second, third, and highest quartiles experienced progressively higher mortality compared with the lowest quartile, with exponentiated coefficients of 1.08 (95% CI 1.04–1.12, *p* < 0.001), 1.10 (95% CI 1.06–1.14, *p* < 0.001), and 1.11 (95% CI 1.07–1.15, *p* < 0.001), respectively.

In contrast, minority status and language vulnerability (RPL_THEME3) were associated with lower mortality in higher quartiles. Significant reductions in mortality were observed for the third and highest quartiles compared with the lowest quartile, with exp(coef) 0.95 (95% CI 0.92–0.98, *p* = 0.002) and 0.93 (95% CI 0.89–0.96, *p* < 0.001), respectively. The second quartile did not differ significantly from the reference.

Socioeconomic vulnerability (RPL_THEME1) and housing type and transportation vulnerability (RPL_THEME4) showed no significant associations across quartiles, with all quartile comparisons yielding coefficients close to unity and non-significant *p*-values. Overall, SVI quartiles similarly demonstrated no significant associations with mortality in univariable models (RPL_THEME).

### 3.5. Multivariable Dose–Response Associations Between SVI Quartiles and RA Mortality

Quartile-based multivariable analyses further clarified these relationships and suggested dose–response patterns across increasing levels of vulnerability (Table 3). Compared with counties in the lowest quartile of household composition and disability vulnerability (RPL_THEME2), progressively higher quartiles were associated with stepwise increases in RA mortality. Counties in the highest quartile demonstrated a 17% higher mortality rate relative to the reference group (Q4 vs. Q1: exp(coef) 1.17, 95% CI 1.12–1.22, *p* < 0.001), with statistically significant increases observed across all intermediate quartiles.

In contrast, higher quartiles of socioeconomic vulnerability were consistently associated with lower mortality (RPL_THEME1). Compared with the least vulnerable counties, mortality rates were lower in counties within the second, third, and highest quartiles of socioeconomic vulnerability, with the strongest association observed in the highest quartile (Q4 vs. Q1: exp(coef) 0.91, 95% CI 0.87–0.95, *p* < 0.001). A similar inverse gradient was observed for minority status and language vulnerability (RPL_THEME3), with significantly lower mortality in the third and highest quartiles, although the second quartile did not differ significantly from the reference group.

The housing type and transportation domain (RPL_THEME4) showed no consistent pattern across quartiles. While the highest quartile was associated with a modest increase in mortality, intermediate quartiles were not significantly different from the reference group, suggesting an inconsistent or weak association.

## 4. Discussion

In this nationwide county-level study covering 2010–2019, overall social vulnerability, as measured by the composite Social Vulnerability Index (SVI), was not significantly associated with RA–related mortality. However, substantial heterogeneity was observed across individual SVI domains. Vulnerability related to household composition and disability demonstrated a consistent positive association with mortality, whereas socioeconomic status and minority status and language showed more complex and context-dependent relationships after multivariable adjustment.

The mortality burden observed in this study should be interpreted alongside prior U.S. and global estimates. In our analysis, 354,280 deaths from 2010 to 2019 were identified among individuals with RA recorded as a cause of death. By comparison, a recent CDC WONDER-based U.S. analysis reported that RA-related mortality declined from 1999 to 2019 but increased again in 2020, suggesting that RA mortality remains a persistent public health concern [5]. At the global level, the GBD 2021 RA study estimated approximately 38,300 deaths due to RA in 2020 [19]. Differences in absolute estimates across studies likely reflect differences in outcome definition and analytic scale, including whether RA was recorded as any cause of death, as in our study, or only as the underlying cause of death. Therefore, direct numerical comparisons should be interpreted cautiously, but these data collectively support RA-related mortality as a continuing global and U.S. public health burden.

Among the four SVI domains, household composition and disability emerged as the most robust and consistent associated factors of RA-related mortality (Table 1, Table 2 and Table 3, Figure 1). Counties with higher proportions of older adults, individuals with disabilities, and single-parent households experienced higher RA mortality rates, with a clear gradient observed across increasing vulnerability quartiles (Table 2 and Table 3). These findings are consistent with prior literature emphasizing the role of functional limitation, disability burden, and social support in determining outcomes in chronic inflammatory diseases [14]. Individuals with RA who experience physical disability may face barriers to timely diagnosis, sustained disease-modifying therapy, and continuity of care. All of the factors are known to adversely affect long-term survival. At the population level, this domain likely captures the combined effects of frailty, comorbidity burden, and reduced caregiving capacity, providing a plausible explanation for its strong association with mortality. The strong association of household composition and disability vulnerability with RA mortality also highlights the potential importance of rehabilitation and functional support. Beyond pharmacologic control of inflammation, multidisciplinary management that includes physical therapy, occupational therapy, assistive devices, fall prevention, and caregiver support may help preserve mobility, reduce disability progression, and improve resilience during acute illness. At the population level, counties with high disability-related vulnerability may benefit from integrated care models that combine rheumatology treatment with rehabilitation services and community-based functional support.

An important observation from this study is the divergence between univariable and multivariable quartile-based analyses for certain SVI domains. For example, socioeconomic vulnerability (RPL_THEME1) did not show a significant association with mortality in univariable quartile-based analyses (Table 2) but appeared inversely associated with mortality after adjustment for other SVI domains in multivariable models (Table 3). This finding suggests that the relationship between socioeconomic disadvantage and RA mortality may be confounded by other dimensions of vulnerability, particularly household composition and disability or minority status and language. In other words, counties with higher socioeconomic vulnerability often differ systematically across multiple social dimensions, and adjustment for these correlated factors may reveal associations that are not apparent in univariable models [20]. These results highlight the interdependence of social determinants and support the use of complementary modeling approaches, including both continuous and categorical analyses, to better delineate the contributions of each domain.

In multivariable quartile-based analyses adjusting for all SVI domains, higher socioeconomic vulnerability was inversely associated with county-level RA mortality after adjustment for the remaining domains, whereas no such association was observed in univariable models. (Table 2 and Table 3). Higher RPL_THEME1 scores (greater socioeconomic disadvantage) were associated with lower RA mortality at the county level. One possible explanation relates to the study period from 2010 to 2019, which coincided with major changes in U.S. health policy, including the implementation and expansion of Medicaid under the Affordable Care Act. These policy changes may have improved insurance coverage and access to care among socioeconomically disadvantaged populations, potentially attenuating traditional socioeconomic gradients in RA outcomes at the population level. However, this interpretation remains hypothesis-generating. Because the present study used county-level ecological data, we could not directly assess individual insurance status, healthcare utilization, rheumatology access, DMARD use, or changes in treatment continuity.

Higher vulnerability related to minority status and language was associated with lower RA mortality in multivariable analyses, a pattern consistent across continuous and quartile-based models (Figure 1 and Table 3). This seemingly appears paradoxical finding with prior literature linking minority populations to adverse health outcomes. Several non-mutually exclusive explanations may contribute. Counties with larger racial and ethnic minority populations are often urban and may have greater proximity to tertiary care centers, safety-net hospitals, and rheumatology specialists, potentially facilitating access to DMARD therapy and specialist care; urban residents with RA are more likely to report rheumatologist care than rural residents [20]. Differential patterns of mortality attribution and case ascertainment may also influence results. RA may be underdiagnosed or underreported as a cause of death in more socioeconomically advantaged or rural counties, whereas urban counties with larger minority populations may have more systematic documentation of chronic inflammatory conditions on death certificates [21]. Because urbanicity was not directly incorporated into the present models, residual confounding by urban–rural context cannot be excluded. Future analyses incorporating rural–urban continuum codes, provider density, and rheumatology access measures may help clarify the extent to which urbanicity modifies or confounds the association between SVI domains and RA mortality. Selective survival may further contribute, as individuals with severe disease in highly vulnerable settings may die from competing causes, attenuating observed RA-specific mortality at the county level.

Our findings should be interpreted in the context of emerging studies linking social vulnerability to mortality in other immune-mediated rheumatic diseases, particularly systemic lupus erythematosus (SLE). In a state- and county-level analysis of SLE-related mortality in the US, Pamuk et al. reported that counties with higher SVI scores had higher SLE-related age-adjusted mortality rates, with a significant correlation between county-level SVI and SLE mortality [22]. They also found that declines in SLE-related mortality over time were less pronounced in regions with higher social vulnerability, suggesting that social vulnerability may influence not only mortality burden but also improvement in mortality trends. In a hospitalized SLE cohort, Kim et al. reported that patients in the highest SVI quartile died at a younger mean age than those in the lowest quartile and that higher SVI was associated with increased risk of death [23]. Similarly, Reed et al. found that neighborhood-level low economic and household instability factors were associated with early mortality among patients with SLE after adjustment for race, sex, and disease severity [24]. Carter et al. also reported that mortality was increased among lupus patients living in socially vulnerable census tracts, particularly among patients with disease damage [25]. Together, these findings indicate that social vulnerability is increasingly recognized as a mortality-relevant contextual factor in SLE. Our study extends this emerging literature to RA and suggests that, while social vulnerability is relevant across immune-mediated rheumatic diseases, the domain-specific mortality patterns may differ by disease, outcome definition, population, and analytic scale. Clinically, these findings reinforce the importance of considering social context when managing RA and other chronic inflammatory diseases. Social vulnerability may influence not only access to diagnosis and specialist care but also treatment continuity, ability to adhere to therapy, functional capacity, and resilience during comorbid illness. Accordingly, patient-centered management should incorporate assessment of functional limitations, caregiving resources, transportation barriers, insurance coverage, and access to rehabilitation or community support services.

Several limitations should be acknowledged. First, this was an ecological study, and associations observed at the county level may not reflect individual-level relationships. Residual confounding by unmeasured factors, including comorbidity burden, disease severity, treatment patterns, and healthcare utilization, cannot be excluded. Second, mortality estimates derived from death certificates are subject to misclassification and variability in reporting across jurisdictions [26]. Third, the study period overlapped with major healthcare policy changes, which may limit generalizability to other periods. Finally, the use of static social vulnerability measures precludes assessment of how changes in vulnerability over time may relate to mortality trends. Despite these limitations, this study has notable strengths, including its national scope, use of standardized mortality metrics, and comprehensive evaluation of both continuous and quartile-based SVI measures. The consistency of findings across multiple analytic strategies strengthens confidence in the observed associations.

## Figures and Tables

**Table 1:** Association between Social Vulnerability Index (SVI) domains and mortality among individuals with RA. Values are presented as exponentiated coefficients with 95% confidence intervals (CI). The CDC SVI includes an overall social vulnerability index (RPL_THEMES) and four domains: socioeconomic status (RPL_THEME1), household composition & disability (RPL_THEME2), minority status & language (RPL_THEME3), and housing type & transportation (RPL_THEME4).

Feature	Coefficient	95% CI	*p* Value
RPL_THEMES	1.000	(0.999, 1)	0.205
RPL_THEME1	1.000	(0.999, 1)	0.208
RPL_THEME2	1.136	(1.09, 1.185)	<0.001
RPL_THEME3	0.895	(0.857, 0.935)	<0.001
RPL_THEME4	1.020	(0.977, 1.066)	0.367

**Table 2:** Univariable Gamma regression results of mortality among individuals with RA across quartiles of Social Vulnerability Index (SVI) subthemes. Compared with the lowest quartile (Q1, reference), higher quartiles of Household Composition & Disability (RPL_THEME2) were consistently associated with greater mortality risk, while higher quartiles of Minority Status & Language (RPL_THEME3) were associated with lower mortality. Socioeconomic Status (RPL_THEME1) and Housing & Transportation (RPL_THEME4) showed no consistent association. Q1 represents the lowest 0–25% vulnerability, Q2 25–50%, Q3 50–75%, and Q4 the highest 75–100%.

Feature	Quartile	Coefficient	95% CI	*p* Value
RPL_THEMES	Q2	0.993	(0.96, 1.028)	0.692
RPL_THEMES	Q3	0.995	(0.962, 1.03)	0.786
RPL_THEMES	Q4	1.008	(0.974, 1.043)	0.656
RPL_THEME1	Q2	0.988	(0.955, 1.023)	0.501
RPL_THEME1	Q3	0.992	(0.958, 1.026)	0.627
RPL_THEME1	Q4	1.015	(0.981, 1.05)	0.381
RPL_THEME2	Q2	1.079	(1.043, 1.116)	<0.001
RPL_THEME2	Q3	1.1	(1.063, 1.137)	<0.001
RPL_THEME2	Q4	1.108	(1.071, 1.145)	<0.001
RPL_THEME3	Q2	0.979	(0.946, 1.013)	0.218
RPL_THEME3	Q3	0.948	(0.917, 0.981)	0.002
RPL_THEME3	Q4	0.925	(0.894, 0.958)	<0.001
RPL_THEME4	Q2	1.012	(0.978, 1.047)	0.488
RPL_THEME4	Q3	0.985	(0.952, 1.019)	0.389
RPL_THEME4	Q4	1.022	(0.988, 1.057)	0.216

**Table 3:** Multivariable Gamma regression results of mortality among individuals with RA across quartiles of Social Vulnerability Index (SVI) subthemes. Compared with the lowest quartile (Q1, reference), higher quartiles of Household Composition & Disability (RPL_THEME2) were consistently associated with greater mortality risk, while higher quartiles of Socioeconomic Status (RPL_THEME1) and Minority Status & Language (RPL_THEME3) were associated with lower mortality. Housing & Transportation (RPL_THEME4) showed no consistent association. Q1 represents the lowest 0–25% vulnerability, Q2 25–50%, Q3 50–75%, and Q4 the highest 75–100%.

Feature	Quartile	Coefficient	95% CI	*p* Value
RPL_THEME1	Q2	0.936	(0.902, 0.971)	<0.001
RPL_THEME1	Q3	0.912	(0.875, 0.951)	<0.001
RPL_THEME1	Q4	0.908	(0.866, 0.952)	<0.001
RPL_THEME2	Q2	1.106	(1.068, 1.146)	<0.001
RPL_THEME2	Q3	1.153	(1.108, 1.199)	<0.001
RPL_THEME2	Q4	1.173	(1.123, 1.224)	<0.001
RPL_THEME3	Q2	0.984	(0.952, 1.018)	0.353
RPL_THEME3	Q3	0.952	(0.92, 0.985)	0.00435
RPL_THEME3	Q4	0.916	(0.883, 0.95)	<0.001
RPL_THEME4	Q2	1.021	(0.986, 1.056)	0.24
RPL_THEME4	Q3	1.008	(0.973, 1.045)	0.65
RPL_THEME4	Q4	1.08	(1.038, 1.123)	<0.001

## Data Availability

The data used in this study are publicly available from the U.S. Centers for Disease Control and Prevention (CDC). These data can be accessed through the CDC Wide-ranging Online Data for Epidemiologic Research (WONDER, https://wonder.cdc.gov/ (accessed on 7 May 2026)) database and the CDC Social Vulnerability Index (SVI) database.

## Supplementary Materials

The following supporting information can be downloaded at: https://www.mdpi.com/article/10.3390/medsci14020314/s1. Table S1. Multicollinearity diagnostics and pairwise correlations among Social Vulnerability Index thematic domains.
