# Supplementary material for: County-Level Association Between Social Vulnerability and Rheumatoid Arthritis-Related Mortality in the United States
Source: Med Sci (Basel). 2026 Jun 12;14(2):314. doi: 10.3390/medsci14020314 (PMC13303296; doi:10.3390/medsci14020314)
Supplement: Supplementary file 1 [file medsci-14-00314-s001.zip › medsci-4278315-supplementary.pdf]

Table S1. Multicollinearity diagnostics and pairwise correlations among Social Vulnerability Index thematic domains.

	RPL_THEME1	RPL_THEME2	RPL_THEME3	RPL_THEME4
**RPL_THEME1**	1	−0.021	−0.029	0.006
**RPL_THEME2**	−0.021	1	−0.007	0.201
**RPL_THEME3**	−0.029	−0.007	1	0.4
**RPL_THEME4**	0.006	0.201	0.4	1
